# Integration of Physical Features and Machine Learning: CSF-RF Framework for Optimizing Ground Point Filtering in Vegetated Regions

**DOI:** 10.3390/s25195950

**Published:** 2025-09-24

**Authors:** Sisi Zhang, Chenyao Qu, Zhimin Wu, Wei Wang

**Affiliations:** School of Geosciences and Info-Physics, Central South University, Changsha 410083, China; zhangsisi_csu@csu.edu.cn (S.Z.); chenyaoqu@csu.edu.cn (C.Q.); wuzhimin@csu.edu.cn (Z.W.)

**Keywords:** vegetated areas, Cloth Simulation Filter (CSF), Random Forest (RF), point cloud filtering, machine learning

## Abstract

Complex terrain conditions and dense vegetation cover in a vegetation area present significant challenges for point cloud data processing and the accurate extraction of ground points. This work integrates the physical characteristics between ground and non-ground points from the traditional Cloth Simulation Filter (CSF) algorithm and the strong learning capability of the machine learning Random Forest (RF) framework, developing the CSF-RF fusion algorithm for filtering ground points in vegetated areas, which can improve the accuracy of point cloud filtering in complex terrain environments. Both type I and type II errors do not exceed 0.05%, and the total error is maintained within 0.03%. Particularly in areas with dense vegetation and severe terrain undulations, the advantages are evident: the CSF-RF algorithm achieves a total error of only 0.19%, representing a 79.6% relative reduction compared with the 0.93% error of the CSF algorithm, while also reducing cases of ground point omission. Thus, it can be seen that the CSF-RF algorithm can effectively reduce vegetation interference and exhibits good stability, providing effective technical support for the accurate extraction of Digital Elevation Models (DEMs) in vegetated areas.

## 1. Introduction

Remote sensing, as an advanced technology for acquiring spatial information, provides the capability to obtain large-scale, multi-temporal, and multi-source data efficiently and has been widely applied in atmospheric monitoring, environmental management, land surface studies, and three-dimensional urban modeling [1,2,3]. Among these applications, point cloud data obtained from airborne and terrestrial LiDAR has become an important data source for detailed topographic representation and object recognition. Ground point filtering is a key step in LiDAR data processing [4,5]. Its main goal is to accurately distinguish ground points from non-ground points from point cloud data [6]. Ground points generally refer to those that represent the actual terrain surface, while non-ground points include point clouds formed by vegetation, buildings, and other objects. Because the original point cloud data obtained by airborne lidar is mixed with a large number of non-ground points, if it is not processed, it will cause significant errors if it is directly used for terrain modeling. At the same time, ground point filtering is the basis for constructing a high-precision digital elevation model (DEM). At the same time, ground point filtering is the basis for constructing a high-precision digital elevation model (DEM). Accurate ground point extraction can ensure the true reflection of the DEM on topographic relief. In addition, high-quality ground point data are critical for applications such as three-dimensional urban modeling, land use/cover classification, flood simulation, and geological disaster monitoring. Effective filtering processing can improve the reliability and efficiency of subsequent data analysis and reduce the noise and deviation caused by non-ground point interference. Therefore, how to achieve efficient and robust ground point filtering has always been one of the core issues in LiDAR data processing research.

Currently, ground point extraction algorithms for point clouds can be broadly categorized into four types: morphology-based methods, slope-based methods, cloth simulation methods, and machine learning-based filtering methods. The first three are generally referred to as traditional ground point extraction algorithms. Traditional filtering methods mostly used geometric rules for ground point extraction. One of these is the Progressive Morphological Filter (PMF) [4], which removes non-ground points step by step through morphological opening operations. It performs well in relatively flat areas, but it often misses ground points or wrongly removes them in steep or vegetated areas [6]. Slope-based filtering methods [7] use elevation differences and slope angles between points to determine ground and non-ground points; however, they have poor adaptability in areas with drastic slope changes or dense vegetation. The Cloth Simulation Filtering (CSF) algorithm [8] simulates the natural draping of cloth over the point cloud to fit the ground surface, making it more adaptable to complex terrains. However, it is still prone to misclassification in areas with dense vegetation, especially when ground points are hidden by low-lying vegetation. This might cause the simulated cloth surface to deviate from the real terrain surface [8]. In summary, it can be seen that the traditional filtering algorithm can effectively extract ground points in specific scenarios, but there are still inherent limitations, such as sensitivity to parameter settings, inability to perform multi-feature fusion, and limited adaptability under diverse terrain and ground cover conditions.

To overcome the limitations of traditional filtering algorithms in terms of accuracy and adaptability under complex terrain and vegetated conditions, researchers have gradually introduced machine learning and deep learning methods. Different from traditional algorithms that rely on geometric rules and empirical parameter settings, these methods can automatically learn feature patterns in point clouds by training a large amount of sample data, thereby significantly improving the adaptability of the algorithm in complex environments. Deep learning algorithms [9,10,11], such as PointNet, PointNet++, and RandLA-Net, can automatically extract multi-level features directly from the original point cloud to achieve end-to-end training and prediction [12,13,14]. However, deep learning methods usually require a large amount of high-quality labeled data for training, which requires high data acquisition and labeling costs [15,16,17]. At the same time, the training and prediction process has a large computational overhead and significant dependence on hardware resources [18].

To give full play to the advantages of traditional filtering algorithms in parameter transparency, high computational efficiency, and stability under small samples, and take advantage of the strong adaptability and classification ability of machine learning and deep learning in complex terrain and multi-dimensional feature processing, researchers have gradually proposed a fusion algorithm that combines traditional methods with machine learning methods [19,20,21,22,23]. For example, Shi et al. [24] employed the IPTD method for initial ground point extraction and subsequently input multi-scale terrain features (e.g., elevation difference, slope, roughness) into a Support Vector Machine (SVM) for secondary classification. Through the ability of SVM to adaptively learn multi-dimensional terrain features and nonlinear classification, this approach effectively addresses the limitations of traditional slope-based filtering methods, which are easily disturbed by abrupt features at building edges and prone to misclassification in irregular terrains such as mountainous or forested areas. However, since the core classifier of this algorithm is an SVM, its efficiency is insufficient for handling large-scale datasets, making it difficult to process point clouds containing billions of points [25]. Zhao et al. [26] extracted altimetric and radiometric features from LiDAR data and point clouds generated by digital photogrammetry, and then input these features into SVM and Random Forest (RF) classifiers to identify ground and non-ground points. The main advantage of this method lies in its ability to adaptively classify points by integrating multidimensional features, which improves recognition accuracy in complex terrains and urban environments, while also providing better nonlinear handling and robustness compared with purely rule-based methods.

To address these challenges, this study innovatively proposes a CSF-RS algorithm, aiming to improve the accuracy and adaptability of ground point extraction in complex terrains. This method integrates the stability of classical filtering algorithms with the capability of machine learning to capture high-dimensional features, thereby establishing a multi-level enhanced classification framework that provides reliable technical support for generating high-precision DEMs and performing land cover classification. Section 2 describes the principles and workflow of the algorithm, and Section 3 presents the detailed results of the proposed method, including quantitative evaluation metrics and visual analysis, and validates its performance under varying terrain conditions. Section 4 systematically compares the proposed method with other traditional and machine learning-based approaches, highlighting their respective advantages and limitations, and discusses potential scenarios where the proposed method may face challenges. Section 5 summarizes the main findings of the study, emphasizing the effectiveness of the proposed method and its contributions to accurate ground point filtering, and outlines directions for future research.

## 2. Methodology and Experimental Data

### 2.1. Experimental Data

**Figure 1:**
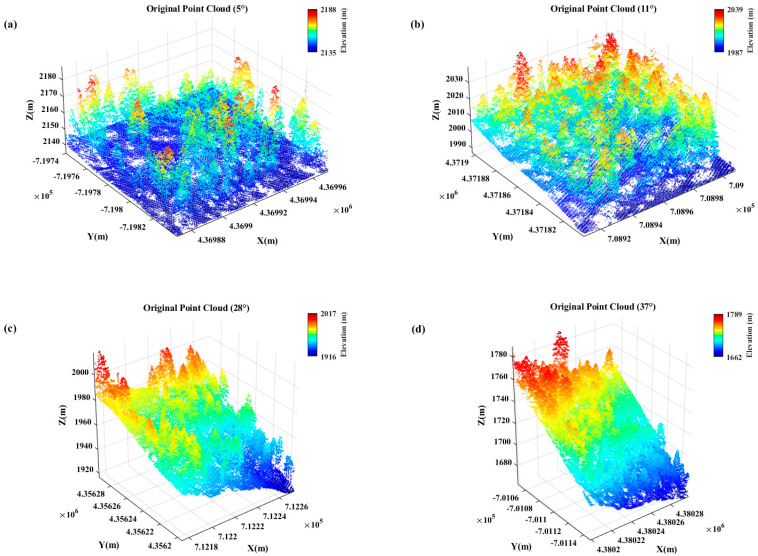
TRI of Sindh Region. Original point cloud data (**a**) 0−5° slope; (**b**) 6−25° slope; (**c**) 25−35° slope; (**d**) >35° slope.

**Figure 2:**
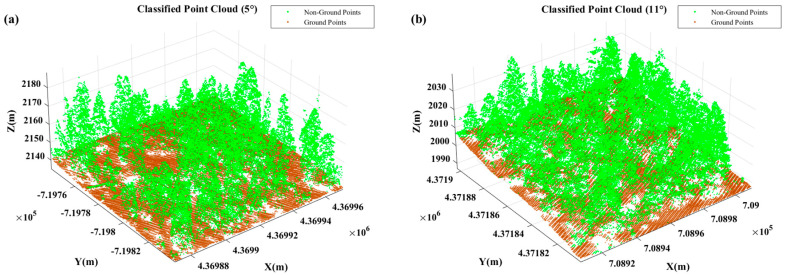

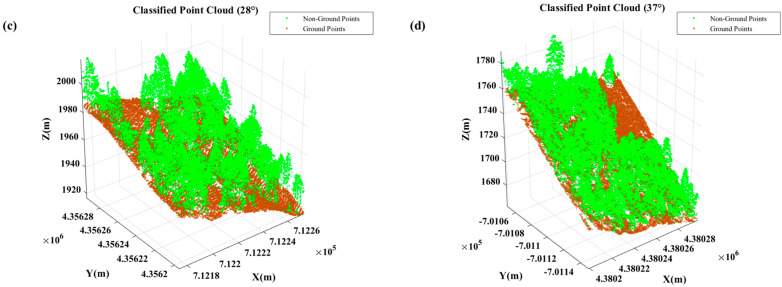
Ground-truth labeling results (**a**) 0−5° slope labels; (**b**) 6−25° slope labels; (**c**) 25−35° slope labels; (**d**) >35° slope labels.

The data used in this study were obtained from the “2014 USFS Tahoe National Forest LiDAR” dataset provided by the Open Topography website. The slope value was calculated as the average slope (in degrees) of all sampling points within each forest stand. According to the *Technical regulations for inventory for forest management planning and design*, slope was reclassified into four categories: flat slope (0–5°), moderate slope (6–25°), steep slope (26–35°), and very steep slope (≥36°). Based on this classification, four-point cloud datasets were selected from vegetated areas (Figure 1), with average slopes of 5°, 11°, 28°, and 37°, respectively, each covering an area of 100 × 100 m. These slope values are consistent with the dominant slope distribution patterns in the broader Tahoe National Forest dataset and thus reflect the typical variability of regional terrain and vegetation conditions. The 100 × 100 m extent was chosen to ensure a sufficient number of LiDAR points for reliable evaluation while maintaining computational efficiency. The ground-truth data for ground point extraction were obtained by performing an initial classification using the Cloth Simulation Filter (CSF) in LiDAR360 V8.0 software, followed by manual corrections based on cross-sectional profiles. Table 1 presents the statistics of ground and non-ground points in the reference datasets, and Figure 2 shows the visualization of the manually labeled results.

### 2.2. CSF-RF Framework

**Figure 3:**
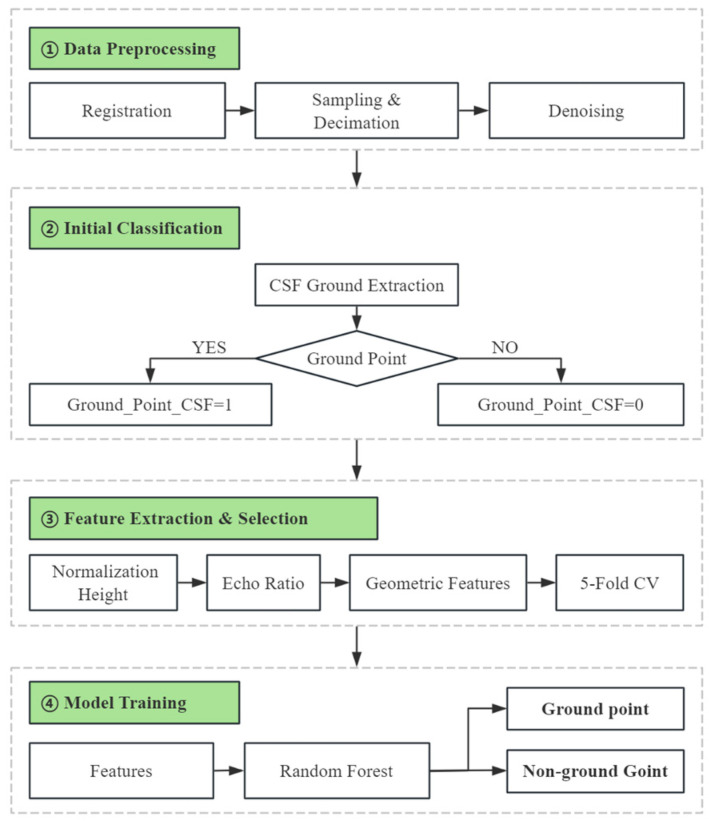
Optimized Point Cloud Filtering Framework.

This study develops a CSF-RF hybrid filtering algorithm for extracting ground points in vegetated areas by integrating the physical modeling capability of the traditional Cloth Simulation Filtering (CSF) algorithm and the strong learning capacity of the Random Forest (RF) machine learning algorithm. The core idea is to first apply CSF for initial ground point extraction and construct a reference surface from the filtered results, which is then used to calculate normalized height values. Multiple point cloud features—such as elevation, density, and curvature—are also extracted as input for Random Forest classification, thereby improving the accuracy of ground point identification. The proposed framework is illustrated in Figure 3.

#### 2.2.1. Point Cloud Data Preprocessing

In order to guarantee the integrity of the point cloud, multiple viewpoints are often used during point cloud data collection. This results in differences in position and orientation between point clouds from different stations. To achieve data uniformity, it is necessary to perform registration on the multi-station point clouds first. Since point cloud datasets are often very large (millions to billions of points), downsampling is necessary to reduce computational load and improve processing efficiency. Additionally, due to the influence of factors such as equipment, surface conditions, and the environment, noise is often present in the raw point cloud. This noise can affect the accuracy of DEM extraction. Therefore, it is necessary to denoise the registered point clouds.

#### 2.2.2. Initial Classification of Point Cloud

The initial classification of point cloud data is mainly carried out using the Cloth Simulation Filtering (CSF) algorithm. This step provides a reference surface for the subsequent fine extraction of ground points. The process includes the following steps: (1) The traditional CSF algorithm is applied to the preprocessed point cloud to extract initial ground points. At this stage, the parameters and thresholds do not need to be very precise—only a rough filtering result is needed. (2) Two attributes are added to each point: whether it is a ground point and the normalized elevation. For all the ground points identified by CSF, set the ground point attribute to 1 and the normalized height to 0. For the other non-ground points identified by CSF, set the ground point attribute to 0, and the normalized height remains unchanged.

#### 2.2.3. Feature Calculation

(1) Normalized Height Calculation

**Figure 4:**
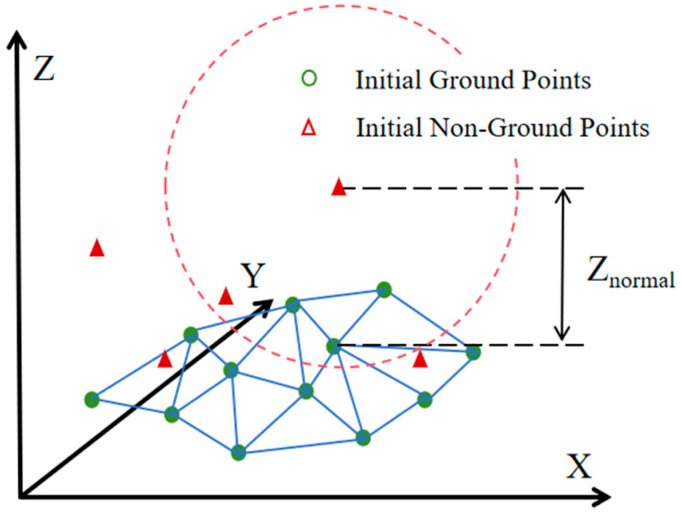
Normalized Elevation Calculation.

The preliminary ground points with a “ground point” attribute value of 0 are extracted from the point cloud. Using a k-d tree search, the nearest point to each of these points is located, and the elevation difference between the two points is assigned as the normalized height value of the nearest point. This value is then written into the “normalized height” attribute of the point cloud, while the preliminary ground points are assigned a “normalized height” attribute value of 0. By repeating the above steps, the normalized height values for all points can be obtained. Figure 4 shows a schematic diagram of the normalized height calculation.

(2) Echo Ratio Calculation

**Figure 5:**
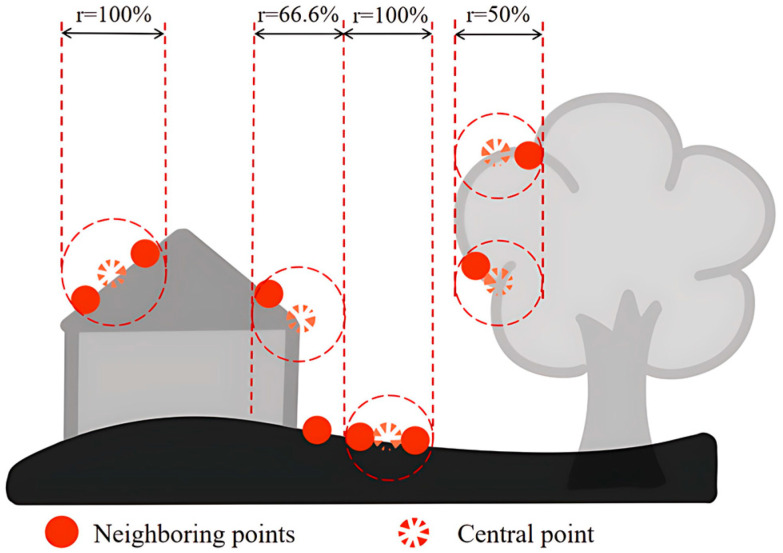
Echo Ratio Calculation.

Modern full-waveform LiDAR systems can record all return signals of a laser pulse, along with the number of returns. The echo ratio is defined as the ratio of the number of points within a radius r around a given point to the total number of points in a vertical column at that location and height range. The calculation formula is shown below. The echo ratio is used as one of the key spatial features in point cloud classification. Figure 5 shows a schematic diagram of the echo ratio calculation.

(1) $$r_{\mathrm{ER}}=\frac{N_{2d}}{N_{3d}}$$

#### 2.2.4. Feature Selection

In the CSF-RF framework, the core machine learning model is the Random Forest (RF) classifier. Experiments show that, compared with Support Vector Machines (SVM), RF is less sensitive to parameter settings and demonstrates greater robustness in processing high-dimensional, multi-feature point cloud data. Its inherent ensemble learning characteristics effectively reduce the risk of overfitting while allowing full utilization of multi-dimensional features for classification and automatic assessment of feature importance. Consequently, RF maintains high classification accuracy even under complex terrain and heterogeneous point cloud conditions. Moreover, RF exhibits high training and prediction efficiency on large-scale datasets, making it particularly advantageous for handling LiDAR point clouds containing hundreds of millions of points.

Effective feature selection during model training is a key factor in ensuring classification performance. The training time is closely related to the number of features—the more features, the longer the training time and the lower the efficiency. This study considers 37 features for selection, including common point cloud features such as scanning information, roughness, curvature, normal vectors, neighboring point statistics, and density, as well as the previously computed normalized height and echo ratio.

In the model evaluation process, we adopted cross-validation. Cross-validation is a commonly used method for assessing generalization performance, which provides a relatively robust error estimate under limited sample conditions [27]. Regarding the choice of the number of folds, previous studies have pointed out that k = 5 or k = 10 are the most common practical options. Among them, ten-fold cross-validation is generally considered to achieve a good balance between bias and variance, while five-fold cross-validation can significantly reduce computational cost while still maintaining reliable estimates [27]. Given the data scale and computational resources in this study, we further compared the performance of five-fold and ten-fold cross-validation. The results showed that five-fold cross-validation achieved an average overall accuracy of 94.14% and an average Kappa coefficient of 88.28%, while ten-fold cross-validation achieved an average overall accuracy of 94.15% and an average Kappa coefficient of 88.30%. This indicates that the differences in accuracy and Kappa coefficient between the two approaches are negligible, whereas five-fold cross-validation demonstrated a clear advantage in computational efficiency (62.34 s vs. 144.64 s). Therefore, we adopted five-fold cross-validation as the evaluation strategy in this study.

The specific steps of five-fold cross-validation for computing the average classification accuracy of the model are as follows: The dataset is randomly divided into five subsets; In each iteration, four subsets are used as the training set to build the Random Forest model; the remaining subset is used as the test set to evaluate the classification performance. At the same time, features with lower average importance are iteratively removed, and the final feature set is selected based on achieving higher overall accuracy (OA) and Kappa coefficient. The feature combinations selected in this study and their average importance are shown in Table 2.

#### 2.2.5. Model Validation and Analysis

The selected 8 features (normalized height, surface roughness, echo ratio, reflection intensity, verticality, eigenvalue ratio, echo beam, and echo number) were input into the random forest model for classification training and cross-validation. The final model achieved an average classification accuracy of 94.16% on the experimental data set. However, this performance result is closely related to the data set used, and its generalization ability still needs to be further verified on other data sets. The filtering accuracy was evaluated using the method proposed by ISPRS. The definitions of filtering errors are provided in Table 3, and the corresponding calculation methods for accuracy metrics are described in Table 4.

Here, TP refers to the number of points correctly classified as ground points after filtering; TN is the number of points correctly identified as non-ground points; FP denotes non-ground points mistakenly classified as ground points; FN represents ground points wrongly classified as non-ground points.

Here, Type I Error refers to the omission rate, where ground points are misclassified as non-ground; Type II Error refers to the commission rate, where non-ground points are misclassified as ground. Total Error indicates the overall proportion of misclassified points. Po is the observed agreement between classification and reference data, while Pe is the expected agreement by chance. Kappa measures the improvement of agreement beyond chance, providing a robust indicator of classification accuracy.

## 3. Results and Analysis

### 3.1. Effectiveness Analysis

(1) Analysis of Feature Selection Effectiveness

During the training of the CSF-RF algorithm model, 8 main features were selected from 37 conventional features, namely normalized_z, scattering, echo_ratio, intensity, verticality, EV_ratio, Number_Of_Returns, and Return_Number. To demonstrate the necessity of feature selection, we compared the filtering accuracy on the test data before and after feature selection. The comparison results are shown in Table 5.

Through comparative analysis before and after feature selection, the CSF-RF method achieved a filtering accuracy of 94.67% and a Kappa coefficient of 89.33% without feature selection, whereas after feature selection, the filtering accuracy was 94.14% and the Kappa coefficient was 88.28%. These results indicate that the differences in model performance before and after feature selection are minor, suggesting that the 29 discarded variables have little impact on the filtering outcome. However, the total runtime of the model was substantially reduced after feature selection, demonstrating that performing feature selection is highly beneficial.

(2) Effectiveness Analysis of Normalized Height Index

The CSF-RF algorithm introduces the normalized height index as one of its key features to enhance the discrimination between ground and non-ground points. To rigorously evaluate the contribution and significance of this normalized height feature, a series of controlled experiments was conducted. Two sets of models were trained: one including the normalized height index as an input feature, and the other excluding it. The performance of these two sets of models was then systematically compared and analyzed. The experimental results are summarized in Table 6.

Further analysis indicates that after adding the normalized height index, the overall accuracy (OA) of the model improved from 88.19% to 94.28%, and the Kappa coefficient also increased from 76.38% to 88.56%, while the total computation time only slightly decreased from 23.13 s to 22.43 s. This result indicates that the normalized height index is highly valuable for improving ground point classification accuracy without significantly increasing the computational burden. After improving feature selection and adding key terrain features, the CSF-RF method has achieved significant improvements in both classification accuracy and computational efficiency, making it highly suitable for processing large-scale point cloud data that requires high-precision filtering.

### 3.2. Results and Accuracy Validation Under Different Terrain Conditions

**Figure 6:**
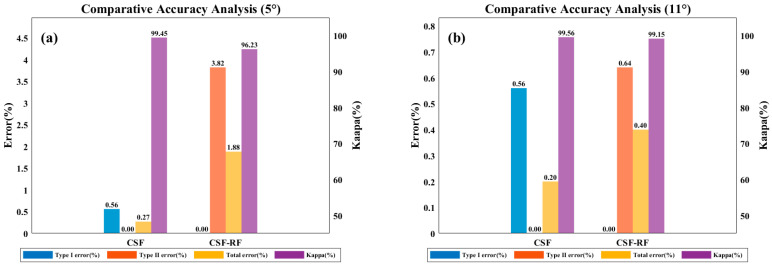

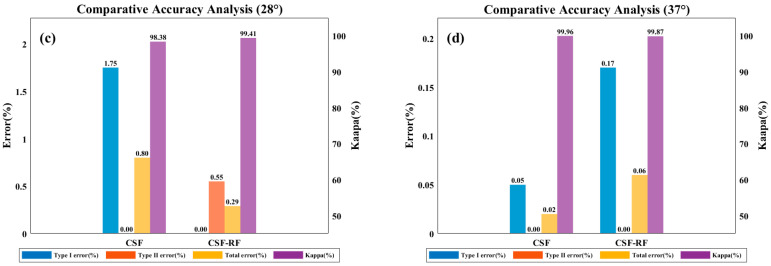
Filtering accuracy of CSF and CSF-RF in diverse terrain scenarios (**a**) 0–5° slope; (**b**) 6–25° slope; (**c**) 25–35° slope; (**d**) >35° slope.

**Figure 7:**
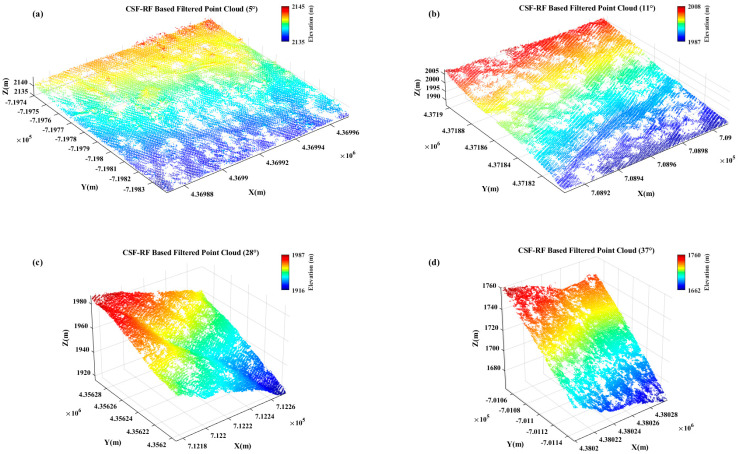
Filtering results of CSF-RF in large-scale complex terrain scenarios (**a**) 0−5° slope; (**b**) 6−25° slope; (**c**) 25−35° slope; (**d**) >35° slope.

Figure 6 and Figure 7 show that for flat slope data, the proposed optimized CSF-RF algorithm has slightly lower accuracy compared to the traditional CSF algorithm, but the overall classification errors are all below 4%, indicating good classification performance. For gentle slope data, the CSF-RF algorithm also performs well and is a suitable choice; for moderate slope data, the CSF-RF algorithm shows better filtering performance on this dataset, with a low type I error and a total error of 0.40%. Its type II error is similar to that of CSF, indicating that the optimized algorithm is also suitable for ground point extraction in moderate slope terrain. For steep slope data, the CSF-RF algorithm outperforms CSF, with a total error of only 0.29% and a type I error of 0.00%. Although the type II error is 0.55%, improvements are seen in total and type I errors. For very steep slope data, the CSF-RF algorithm performs excellently, with a total error of only 0.06%, type I error of 0.17%, and type II error of 0.00%. All error metrics are better than those of the CSF algorithm, making it the optimal method.

It can be seen that the CSF-RF algorithm has significant advantages in ground point classification under common terrain conditions, especially when the slope is steep and sudden, where it is the best option. This method combines the physical simulation robustness of CSF with the discriminative power of Random Forest, ensuring low omission rates while effectively controlling false positives. To further highlight the advantages of the algorithm, this study also tested it on large-scale point cloud datasets collected in dense vegetation scenes, including forested areas with steep slopes and irregular terrain surfaces.

### 3.3. Results and Accuracy Validation in Dense Vegetation Scenes

**Figure 8:**
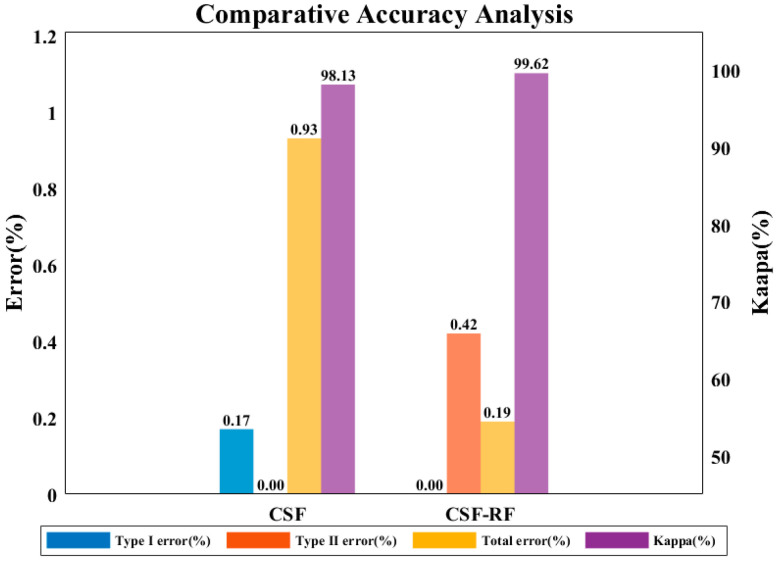
Filtering accuracy of CSF and CSF-RF in dense vegetation scenes.

**Figure 9:**
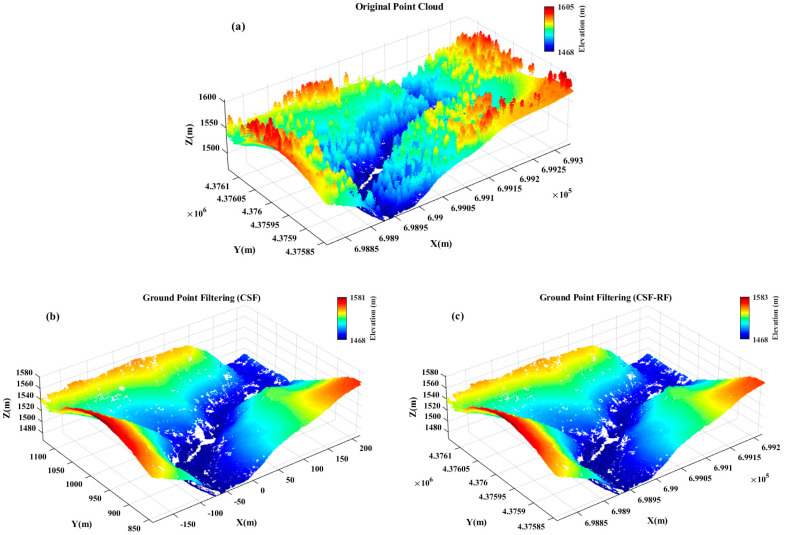
Filtering results of CSF and CSF-RF in dense vegetation scenes. (**a**) Original point cloud; (**b**) CSF filtering result; (**c**) CSF-RF filtering result.

As can be seen from Figure 8 and Figure 9, in dense vegetation scenes, the CSF-RF algorithm shows clear advantages compared to the traditional CSF algorithm. Its key strength lies in reducing type I errors, i.e., the omission rate of ground points, while the CSF-RF algorithm achieves a total error of only 0.19%, representing a 79.6% relative reduction compared with the 0.93% error of the CSF algorithm. Although type II error emerged at 0.42%, the Random Forest model effectively corrected this, maintaining a high Kappa consistency and significantly improving ground point completeness in dense vegetation scenes. Thus, the algorithm is particularly well-suited for high-precision DEM generation and other applications where accurate ground point retention is critical.

## 4. Discussion

### 4.1. Comparison with Other Methods

**Figure 10:**
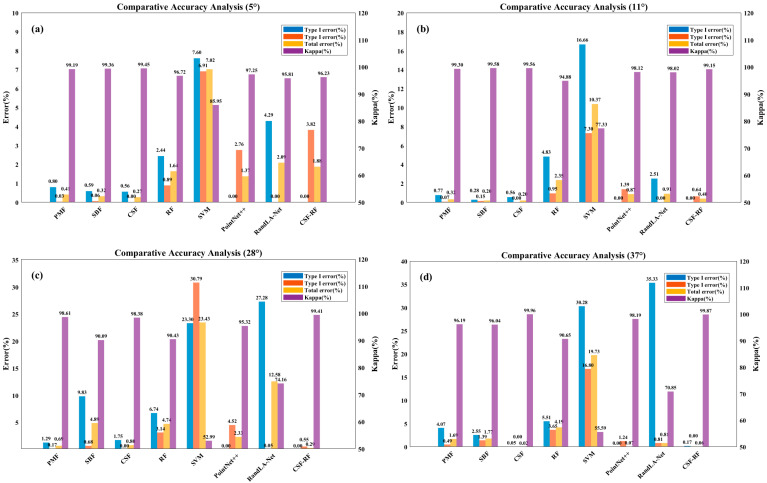
Filtering accuracy analysis in diverse terrain scenarios (**a**) 0–5° slope; (**b**) 6–25° slope; (**c**) 25–35° slope; (**d**) >35° slope.

To test the differences between this algorithm and other traditional algorithms, this study systematically evaluates eight ground point classification algorithms. These include traditional filtering algorithms such as Progressive Morphological Filter (PMF), Slope-Based Filter (SBF), and Cloth Simulation Filter (CSF), as well as machine learning and deep learning algorithms, including Random Forest (RF), Support Vector Machine (SVM), PointNet++, RandLA-Net, and the CSF-RF algorithm. The performance of each algorithm on four types of slopes is compared. Detailed comparisons of the experimental results of each method can be found in Figure 10.

Experimental results show that the CSF-RF hybrid algorithm demonstrates significant technical advantages and practical value: The total error remains strictly below 0.03, with the maximum error not exceeding 0.05, showing excellent stability across different terrain types. Both type I and type II errors remain under 0.05%. In steep and very steep terrain scenarios, its classification accuracy reaches 99.41% and 99.87%, respectively, which is an improvement of 1.03% and 0.91% over the next best-performing CSF algorithm.

In contrast, other algorithms have clear limitations: Traditional PMF and SBF algorithms perform reasonably well in gentle terrain, but their accuracy drops significantly on steep slopes. The machine learning-based RF algorithm demonstrates moderate overall performance, but its accuracy fluctuates greatly in complex terrains. The SVM algorithm performs the worst in all test scenarios. Among deep learning methods, PointNet++ shows good performance in gentle terrain but still has a 4.52% misclassification rate in steep slope scenarios. RandLA-Net experiences severe misclassifications in both steep and very steep terrain, with a maximum misclassification rate reaching 35.33%.

In the extraction of ground points in complex terrains and vegetated areas, various improved algorithms have been proposed in recent years, and their performance comparison is shown in Table 7. The CAP [28] algorithm shows high accuracy in complex urban environments by integrating CSF and progressive TIN encryption modeling. Especially in the building area, it can effectively deal with the interference caused by building edges and abrupt structures. The overall accuracy reaches 97.14%, showing significant advantages. In contrast, in the vegetation-covered area, due to the fact that low vegetation and other features are easily confused with the terrain, some feature points may be misclassified as ground points, so there is still room for further improvement in the adaptability of this type of area. The Shi [24] method employs an improved progressive triangulated irregular network filtering to extract ground points and applies a support vector machine for object classification. It achieves high accuracy in both urban building and vegetation areas (93.3% and 91.6%, respectively), with strengths in automation and robust classification, though it remains sensitive to feature selection and parameter settings. Wu [29] et al. proposed a filtering method for forest areas based on local minima and machine learning, which performs well in complex woodland environments, with an overall accuracy of 87.25% in vegetation regions. This method shows good terrain adaptability and requires fewer parameters, but its performance is still affected by complex canopy structures.

In contrast, the CSF-RF algorithm proposed in this paper maintains the high accuracy of the traditional CSF method while significantly enhancing robustness in complex terrains through feature fusion and classifier optimization. On steep slopes, the classification accuracy improves by 9.98% compared to the original RF. It consistently maintains a high classification accuracy in all test scenarios, providing an innovative solution for terrain classification in complex environments with both high precision and strong stability.

### 4.2. Limitations of the Method

Experiments demonstrate that the CSF-RF algorithm achieves high filtering accuracy and strong terrain adaptability across multiple test datasets, with its effectiveness mainly attributed to the complementary strengths of its two components. The CSF algorithm provides robust terrain modeling and adaptability to varying slope conditions, while the Random Forest classifier is able to fully exploit multi-dimensional geometric and radiometric features for accurate ground/non-ground separation. Based on the experimental results, this hybrid strategy significantly reduces type I errors compared with CSF alone, while also achieving a lower overall error rate than traditional filtering methods, thereby exhibiting greater effectiveness under diverse terrain conditions.

However, some limitations remain. First, the current training of the CSF-RF algorithm primarily relies on specific datasets, and its adaptability and scalability in more diverse scenarios—such as different vegetation types, acquisition conditions, and large-scale datasets—have not yet been fully validated. Second, since the performance of traditional ground point filtering algorithms often depends on parameter settings, this study has not yet examined the impact of CSF preprocessing parameter configurations on the overall performance of the proposed algorithm. Finally, due to the relatively high computational complexity of the Random Forest algorithm in high-dimensional feature spaces, the computation time increases significantly. Therefore, the CSF-RF algorithm still faces challenges related to high computational cost, parameter sensitivity, and uncertain scalability, indicating that further optimization and validation are required.

## 5. Conclusions

In this study, a “CSF-RF” algorithm is proposed by integrating the physical characteristics of ground and non-ground points from the traditional Cloth Simulation Filtering (CSF) algorithm with the powerful learning capability of the Random Forest (RF) algorithm. Experimental results demonstrate that, compared with traditional filtering methods, this approach not only significantly reduces parameter dependency and minimizes the need for manual intervention, but also exhibits strong robustness and stability under different terrain conditions (with all types of errors below 0.05%). In complex forested areas, the method outperforms traditional approaches in terms of filtering accuracy and terrain adaptability (with all types of errors below 0.2%), showing substantial effectiveness and promising application potential in digital elevation model (DEM) construction, forest resource surveys, and ecological environment monitoring.

Future research will further analyze the transferability of the model across datasets with differences in vegetation types, terrain complexity, and acquisition conditions to ensure its robustness in diverse application scenarios. In terms of methodological optimization, more lightweight machine learning models can be considered to reduce computational complexity while integrating CUDA technology and GPU-based parallel computing to enhance computational efficiency. In addition, future work will explore combining random forests with deep learning approaches for hybrid feature extraction, thereby leveraging the strengths of both methods to further reduce the misclassification of near-ground points and maintain stable filtering performance in increasingly complex environments.

## Figures and Tables

**Table 1:** Point Cloud Dataset of Different Slope Grades in Vegetation Zones.

Slope Grade	0–5°	6–25°	25–35°	>35°
Ground Points (count)	42,401	34,762	59,397	43,167
Non-Ground Points (count)	44,061	61,200	69,715	85,222
Total Points (count)	86,102	95,962	129,112	128,389

**Table 2:** Feature Combination Average Importance Ranking Table.

Mean Importance Ranking	Feature	Mean Importance
1	Normalized_z	0.514202
2	Scattering	0.192790
3	Echo_ratio	0.101099
4	Intensity	0.071659
5	Verticality	0.047164
6	EV_ratio	0.034770
7	Number_Of_Returns	0.022380
8	Return_Number	0.015936

**Table 3:** Definition of filtering error.

Category	Ground Points	Non-Ground Points	Total Number
Ground points	TP	FN	TT = TP + FN
Non-ground points	FP	TN	FF = FP + TN
Total number	PP = TP + FP	NN = TN + FN	T = TP + TN + FP + FN

**Table 4:** Calculation methods for accuracy metrics.

Category	Ground Points
Type I Error	FN/(TP + FN)
Type II Error	FP/(FP + TN)
Total Error	(FP + FN)/T
P_o_	(TP + TN)/T
P_e_	((TP + FN)(TP + FP) + (FP + TN)(FN + TN))/T^2^
Kappa	(P_o_ − P_e_)/(1 − P_e_)

**Table 5:** Comparison of filtering accuracy before and after feature selection.

Parameter	Without Feature Selection	After Feature Selection
OA (%)	94.67	94.14
Kappa (%)	89.33	88.28
Model runtime (s)	263.42	68.43

**Table 6:** Comparison of filtering accuracy with and without normalized elevation index selection.

Parameter	With NEI	Without NEI
OA (%)	94.28	88.19
Kappa (%)	88.56	76.38
Total Time(s)	22.43	23.13

**Table 7:** Comparison of ground filtering algorithms.

Reference	Algorithm	Scenarios	OA (%)
Liang [28]	CAP	Building	97.14
Shi [24]	IPTD + SVM	Vegetation	91.60
		Building	93.30
Wu [29]	-	Vegetation	87.25
Proposed algorithm	CSF_RF	Vegetation	94.28

## Data Availability

All data sets of this experiment can be found from Open Topography (https://doi.org/10.5069/G9V122Q1; accessed on 5 March 2025). We express our sincere gratitude to the data providers and related agencies.
